# A Case of Eosinophilic Granulomatosis with Polyangiitis Complicated with A IgG4 Related Disease Like Symptoms

**DOI:** 10.1155/2018/3763084

**Published:** 2018-11-04

**Authors:** Suguru Sato, Julia Morimoto, Yasuharu Oguchi, Takashi Umeda, Takaya Kawamata, Mami Rikimaru, Tatsuhiko Koizumi, Ryuichi Togawa, Yasuhito Suzuki, Yuki Sato, Manabu Uematsu, Hiroyuki Minemura, Takefumi Nikaido, Atsuro Fukuhara, Junpei Saito, Kenya Kanazawa, Yoshinori Tanino, Mitsuru Munakata, Yoko Shibata

**Affiliations:** ^1^Department of Pulmonary Medicine, School of Medicine, Fukushima Medical University, Fukushima, Japan; ^2^Department of Ophthalmology, School of Medicine, Fukushima Medical University, Fukushima, Japan; ^3^Department of Infectious Disease and Pulmonary Medicine, Aizu Medical Center, Fukushima Medical University, Aizuwakamatsu, Japan

## Abstract

We report a case of eosinophilic granulomatosis with polyangiitis (EGPA) complicated with a IgG4 related disease like symptoms presenting as eyelid swellings. In the present case, the serum level of IgG4 and the ratio of IgG4 to IgG were generally increased by the disease course of EGPA. Considering the course of clinical symptoms, there is a possibility that orbital manifestations were one of the clinical features during the disease course of EGPA while the histological features of right eyelid tissue and other ocular manifestations were consistent with the diagnosis of IgG4 related disease.

## 1. Introduction

Eosinophilic granulomatosis with polyangiitis (EGPA) is characterized by severe asthma, blood and tissue eosinophilia, and antineutrophilic-cytoplasm antibody (ANCA) targeting neutrophil myeloperoxidase in only a subset of patients. EGPA is classified among small-vessel vasculitides associated with ANCAs and the hypereosinophilic syndrome and the pathological findings are consisting of necrotizing vasculitis, eosinophilic infiltration, and extravascular granuloma formation. IgG4 related disease (IgG4-RD) is a fibro-inflammatory condition characterized by sclerosing lesions in extranodal sites, lymphadenopathy, and elevated serum IgG4 [1]. Histopathologic features include dense lymphoplasmacytic infiltrates rich in IgG4-positive plasma cells and sclerosing fibrosis. It has been reported that a broad spectrum of clinicopathological features and various clinical phenotype and the disease have many similarities to some forms of systemic vasculitis and granulomatous diseases. From recent reports, serum IgG4 levels are markedly elevated in active Churg-Strauss syndrome (CSS) and correlate with the number of organ manifestations and disease activity [2]. The increased IgG4 production is much more pronounced in CSS than in other disease with granulomatosis with polyangiitis (GPA) or atopic asthma. We describe a case of IgG4 related disease presenting as eyelid swellings during the disease course of EGPA.

## 2. Case Presentation

**Figure 1:**
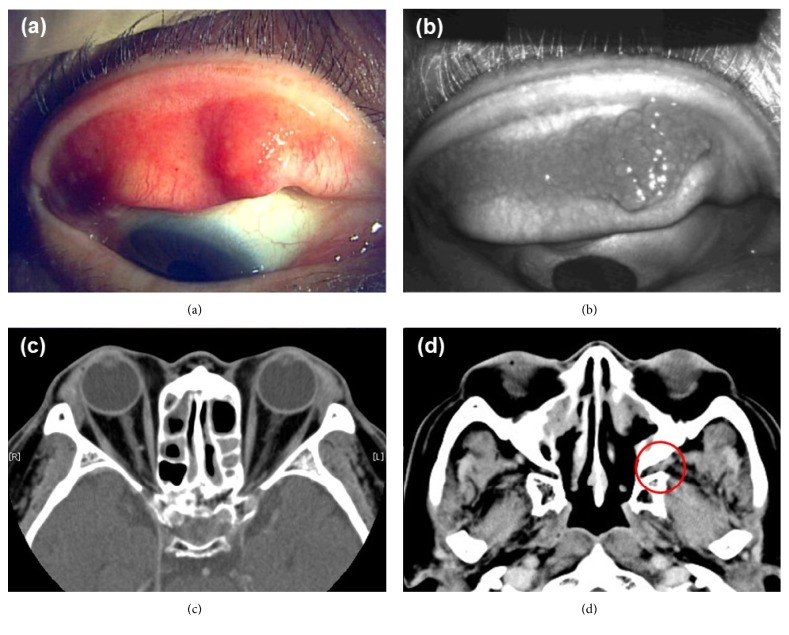
Clinical examination and computed tomography (CT). (a) Color photo and (b) scanning laser ophthalmoscope image showing papillary of right upper eyelid. (c) Orbital CT showing mild swelling of bilateral lacrimal glands. (d) Orbital CT showing hypertrophy of soft tissue in left pterygopalatine fossa (red circle: left pterygopalatine fossa).

**Figure 2:**
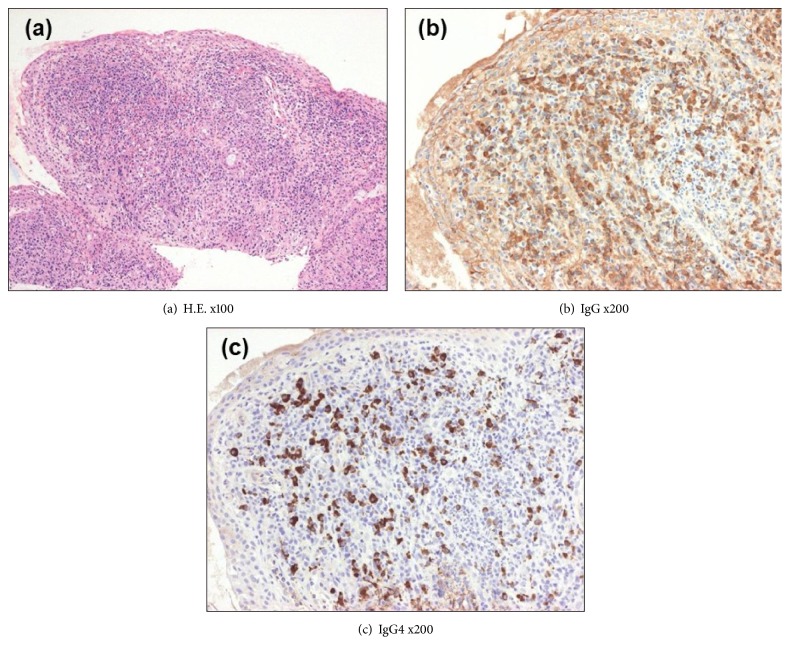
Histopathologic and immunohistochemical findings of right upper eyelid. (a) Histopathologic findings of right upper eyelid included lymphoplasmic infiltration with lymphoid follicles formation consistent with sclerosing inflammation (H & E, x100). (b) Immunostaining identified IgG-positive plasma cells in lymphoid follicle (immunostaining, x200). (c) Immunostaining identified numerous IgG4-positive plasma cells, accounting for 50% of IgG-positive plasma cells (immunostaining, x200).

**Figure 3:**
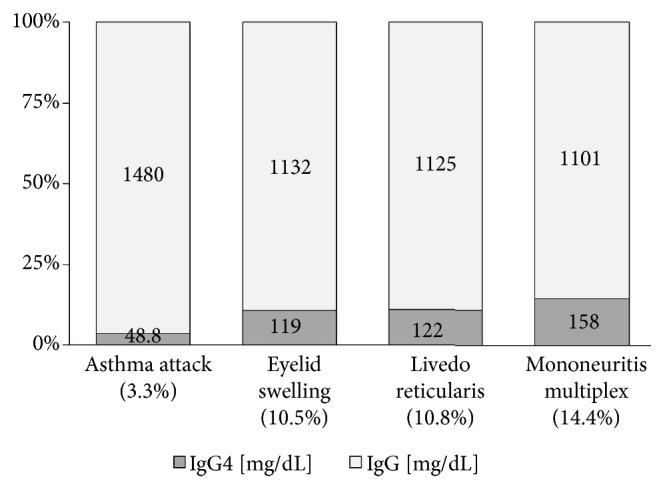
The relationship between clinical symptoms of EGPA and serum level of IgG4 or the ratio of IgG4 to IgG levels.

On April 2011, a 62-year-old male patient presented recurrent wheezes and he was diagnosed with bronchial asthma. He was treated with high dose of inhaled corticosteroids, long acting *β*_2_ agonist, theophylline, leukotriene receptor antagonist, and anti-IgE monoclonal antibody. However, he often suffered from asthma attacks. One year later, laboratory data revealed hypereosinophilia (blood eosinophil count was 1584 per *μ*L) and serum concentration of myeloperoxidase anti-neutrophil antibody (MPO-ANCA) was elevated at the level of 102 U per mL. For treatment of asthma symptom, he was treated with oral prednisone 30 mg per day from June 2012. We observed an improvement of the asthma control with a rapid decrease of serum concentration of MPO-ANCA at the level of 15.9 U per mL on October 2012. The prednisolone was tapered, and he was on 10 mg per day of prednisolone from May 2013. On August 2013, the patient presented the discomfort of bilateral eyelids and papillary swelling of upper eyelids was observed (Figures 1(a) and 1(b)). The computed tomography (CT) image showed bilateral lacrimal gland swellings and hypertrophy of soft tissue in left pterygopalatine fossa (Figures 1(c) and 1(d)). The serum level of IgE, MPO-ANCA, and IgG4 (ratio of IgG4 to IgG) was 237 IU per mL, 21.4 U per mL, and 119 mg per dL (10.5%). Peripheral blood eosinophil count was elevated at the number of 847 per mm^6^. A resection of right eyelid was performed. Histology showed a dense lymphoplasmacytic infiltration with lymphoid follicle formation. In immunostaining for IgG and IgG4 plasma cell, the ratio of IgG4 to IgG was 50% (Figure 2). His serum IgG4 level was 119 mg per dL. After cryothermy coagulations were performed for both eyelid swellings, he had no relapse. No other organ manifestations were found in systemic computed CT for evaluating the progression of IgG4-RD except orbital findings. On May 2014, he felt a pain and revealed livedo reticularis of both legs. Blood eosinophil counts was increased (2260 per *μ*L). A skin biopsy was performed and histology showed perivascular eosinophilic infiltration and deposition of multinucleated giant cells and degenerated eosinophils. The symptom of both legs was caused to the peripheral nerve involvement by mononeuritis multiplex. He was diagnosed as EGPA and treated with steroid pulse therapy. After treatment, the symptom was improved, and blood eosinophil count was decreased to 330 per *μ*L. In this case, serum IgG and IgG4 levels were measured from the first visit (Figure 3). The serum level of IgG4 and the ratio of IgG4 to IgG were generally increased by the disease course of EGPA. He was followed by oral prednisolone at the dose of 40 mg per day and tapered gradually. Two months later, he was started on intravenous cyclophosphamide therapy for tree times. Additionally, high-dose intravenous immunoglobulin was administered for a residual peripheral neuropathy. He subsequently received oral prednisolone at the dose between 15 to 20 mg per day as maintenance therapy. He was doing well as of January 2018.

## 3. Discussion

The present report describes a case of an EGPA concomitant with IgG4 related disease like symptoms during disease course of it. EGPA typically developed into systemic symptoms caused by necrotizing vasculitis such as skin manifestations and peripheral neuropathy followed by allergic symptoms such as bronchial asthma, sinusitis, and allergic rhinitis. In the present case, eyelid swellings and other orbital manifestations were emerged after onset of asthmatic symptoms. There are past reports about orbital manifestations about patients with systemic necrotizing vasculitides. In the past study of identified 270 patients with EGPA, 30 (11%) had any ophthalmologic manifestations at diagnosis and 2 (0.7%) had orbital inflammatory disease [3]. One of the ocular manifestations in CSS was orbital inflammatory pseudotumor [4] and EGPA is a differential diagnosis of IgG4-RD. Ferry et al. reported the retrospective evaluation about the applicability to IgG4-RD diagnostic criteria for previously diagnosed orbital inflammatory pseudotumor or chronic dacryoadenitis [5]. In their report, 15 of 38 cases fulfilled the criteria of IgG4-RD and no patients with EGPA were included the cases, while 3 cases with GPA were included in the inflammatory pseudotumor groups without representing IgG4-RD. In the cases with diagnosis of GPA, increased IgG4-positive plasma cell in orbital biopsies was reported [2, 6]. There are some reports about CSS concomitant with clinical conditions similar to IgG4-RD [7, 8]. However, reports about the orbital manifestations similar to IgG4-RD in EGPA were relatively rare [9].

In a recent report, the possibility of interaction between IgG4-RD and allergic disorders has been advocated [2, 10]. In our case, considering the course of clinical symptoms, there is a possibility that orbital manifestations were one of the clinical features during the disease course of EGPA while the histological features of right eyelid tissue and other ocular manifestations were compatible with the diagnosis of IgG4-RD. We have to consider the possibility of various manifestations appearing in the clinical course of EGPA having crucial relevance with IgG4. Further study is needed to confirm our experience in the careful pathologic evaluation of orbital manifestations in EGPA, and further study of potential relationship between EGPA and IgG4-RD might contribute to elucidate the mechanisms of both disorders.

## Abbreviations

EGPA: Eosinophilic granulomatosis with polyangiitis
ANCA: Antineutrophilic-cytoplasm antibody
IgG4-RD: IgG4 related disease
CSS: Churg-Strauss syndrome
GPA: Granulomatosis with polyangiitis
MPO-ANCA: Myeloperoxidase anti-neutrophil antibody.
